# Inflammasome-targeting natural compounds in inflammatory bowel disease: Mechanisms and therapeutic potential

**DOI:** 10.3389/fimmu.2022.963291

**Published:** 2022-08-24

**Authors:** Qiuyun Xu, Weichen Sun, Jie Zhang, Youmin Mei, Jingyin Bao, Shengping Hou, Xiaorong Zhou, Liming Mao

**Affiliations:** ^1^ Department of Immunology, School of Medicine, Nantong University, Nantong, China; ^2^ Department of Periodontology, Nantong Stomatological Hospital, Nantong, China; ^3^ Basic Medical Research Center, School of Medicine, Nantong University, Nantong, China; ^4^ The First Affiliated Hospital of Chongqing Medical University, Chongqing Eye Institute, Chongqing Key Laboratory of Ophthalmology, Chongqing, China

**Keywords:** traditional medicine, inflammatory bowel disease, NLRP3, inflammasome, IL-1β, natural compounds

## Abstract

Inflammatory bowel disease (IBD), mainly including Crohn’s disease and ulcerative colitis, seriously affects human health and causes substantial social and economic burden. The pathogenesis of IBD is still not fully elucidated, whereas recent studies have demonstrated that its development is associated with the dysfunction of intestinal immune system. Accumulating evidence have proven that inflammasomes such as NLRP3 and NLRP6 play a prominent role in the pathogenesis of IBD. Thus, regulating the activation of inflammasomes have been considered to be a promising strategy in IBD treatment. A number of recent studies have provided evidence that blocking inflammasome related cytokine IL-1β can benefit a group of IBD patients with overactivation of NLRP3 inflammasome. However, therapies for targeting inflammasomes with high efficacy and safety are rare. Traditional medical practice provides numerous medical compounds that may have a role in treatment of various human diseases including IBD. Recent studies demonstrated that numerous medicinal herb derived compounds can efficiently prevent colon inflammation in animal models by targeting inflammasomes. Herein, we summarize the main findings of these studies focusing on the effects of traditional medicine derived compounds on colitis treatment and the underlying mechanisms in regulating the inflammasomes. On this basis, we provide a perspective for future studies regarding strategies to improve the efficacy, specificity and safety of available herbal compounds, and to discover new compounds using the emerging new technologies, which will improve our understanding about the roles and mechanisms of herbal compounds in the regulation of inflammasomes and treatment of IBD.

## Introduction

Inflammatory bowel disease (IBD), mainly including Crohn’s disease and ulcerative colitis, is a term to describe a group of chronic relapsing inflammatory disorders occurred in the gastrointestinal (GI) tract (1–3). These diseases are associated with high risks to develop colorectal cancer, thus extremely affect people’s health and cause serious social and economic burden (4–6). Recent advances have shown that the development of IBD is owing to an aberrant and persistent immune responses to the commensal microflora in the intestine and colon of genetically susceptible individuals (3), whereas the exact pathogenesis of these diseases is still not fully elucidated. The entry of luminal organisms or their products to the lamina propria can be sensed by the tissue resident immune cells such as macrophages and dendritic cells, which process and present antigens to facilitate the activation of adaptive immunity. Meanwhile, the activation of these cells may produce various inflammatory mediators, such as pro-inflammatory cytokines and chemokines, contributing to the amplification of immune responses in the lamina propria of the GI tract.

**Figure 1:**
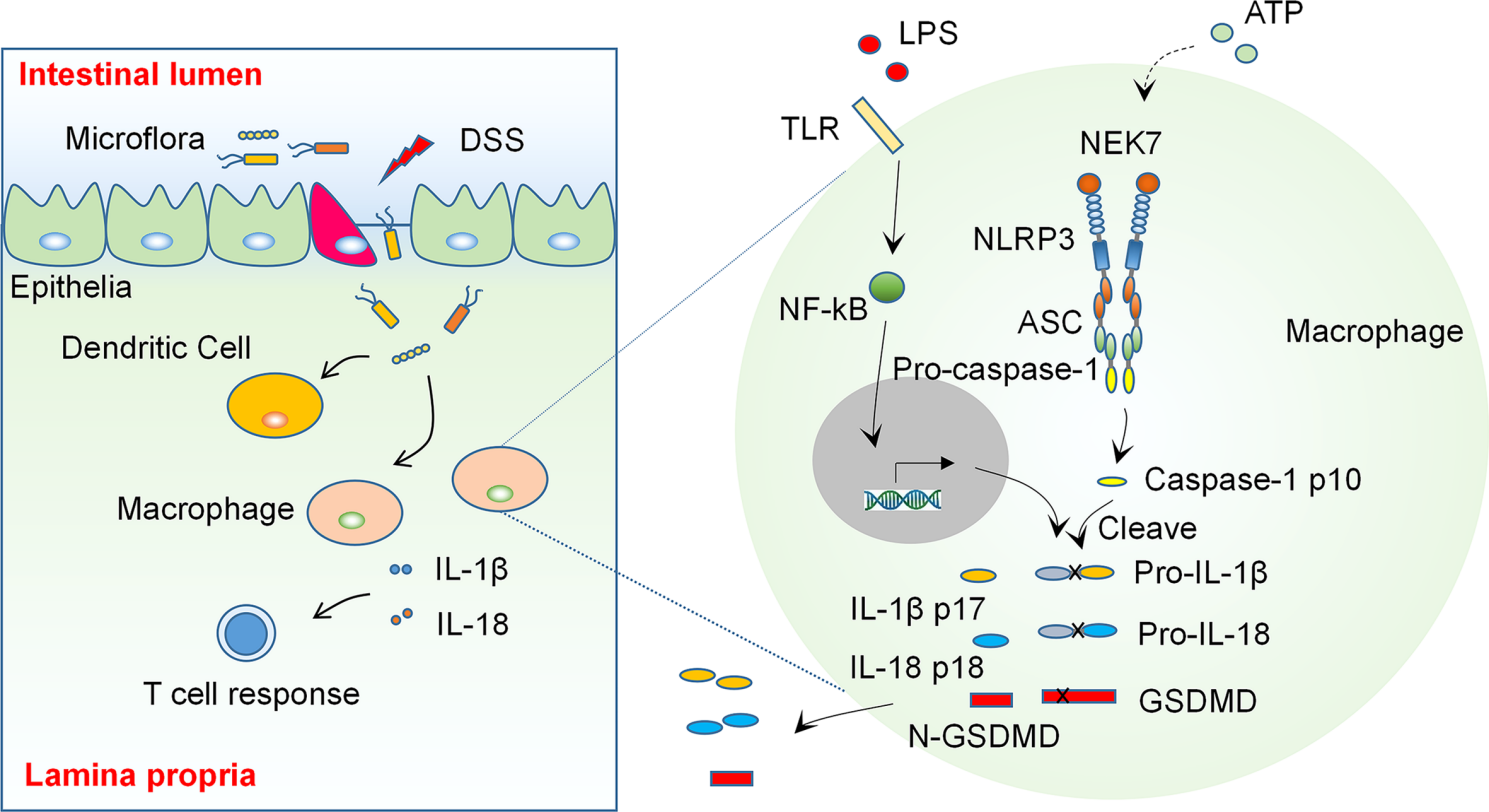
The NLRP3 Inflammasome is an Essential Regulator in the Pathogenesis of Inflammatory Bowel Disease. Some environmental factors cause epithelial damage, which allows the entry of luminal organisms or their products to the lamina propria, the latter then induces activation of inflammasomes and production of pro-inflammatory cytokines such as IL-1β and IL-18 in colon macrophages. These cytokines regulate the adaptive immunity and cause an imbalanced inflammatory response and tissue damage in genetically susceptible individuals (Left). The activation of NLRP3 inflammasome needs two signals: A stimulation to TLRs or TNF receptors induces transcription and appropriate post-translational modification of NLRP3 inflammasome components (signal 1). Some extracellular stimulants such as ATP induce inflammasome assembly (signal 2) and cause autocleavage of caspase-1, which then catalyzes the maturation of precursor of IL-1β, IL-18 and GSDMD, the latter then regulate the adaptive immunity and cause pyroptosis.

As prominent mediators of inflammatory response, the inflammasomes are considered to be critical regulators in the development of IBD. Recent studies have demonstrated that the activation of inflammasomes play an essential role in the pathogenesis of IBD (7). The aberrant activation of inflammasomes and the resultant production of pro-inflammatory cytokines in colonic macrophages may lead to an imbalanced inflammatory response and cause tissue damage in the colon, which may trigger the onset of IBD (see Diagram in **Figure 1**). Therefore, targeting inflammasomes may be a promising strategy for treatment of IBD. A growing body of studies have shown that targeting IL-1β, a downstream cytokine activated by inflammasomes, using neutralizing antibodies is effective in ameliorating colonic inflammation in both animal models of colitis and IBD patients (8), whereas the high cost of antibody based therapy may limit its application (9). Some emerging natural compounds derived from traditional medicine may provide new options for IBD intervention *via* modulating inflammasomes and have been shown to be effective in an increasing number of studies (10–13). This review aims at summarizing these studies regarding the role of ingredients or natural compounds from traditional medicinal herbs in colitis and related mechanisms, and providing an outlook for future studies on investigation of IBD targeting therapies.

## Inflammasomes are critical regulators of IBD

Inflammasomes are a group of large molecular weight multiprotein complexes formed by a sensor protein of the NOD-like receptor family or HIN-200 family, the adaptor protein ASC and the effector caspase-1 in response to stimulation of microbial or damage associated molecular patterns (14, 15). Upon activation, inflammasome formation allows autocatalytic cleavage of caspase-1, which then processes precursors of IL-1β and IL-18 to allow the release of mature cytokines from cells, which may regulate Th17 and Th1 cells, respectively, and amplify the immune responses in the tissue. Meanwhile, the activation of caspase-1 induces a form of programmed cell death, pyroptosis (16–18), which regulates the progression of a variety of inflammatory diseases, including IBD.

To date, a number of PRR sensor proteins, including NLRP3, NLRP1, NLRC4, AIM2 and Pyrin, have been confirmed to form inflammasomes and regulate the activation of caspase-1 by many studies. Moreover, many other PRR sensors, mainly including NLRP2, NLRP6, NLRP7, NLRP9 (19, 20), NLRP12 and IFI16, have also been reported to have inflammasome-forming ability, although these findings need to be further confirmed by other researchers.

NLRP3 is one of the most studied NOD-like receptors that can form an inflammasome. The activation of NLRP3 needs two kinds of stimuli (see Diagram in **Figure 1**): The first one may derive from extracellular or intracellular receptors, such as TLRs or TNF receptors. This stimulus triggers the transcription of inflammasome associated genes and also induces appropriate post-translational modifications of the translated proteins, which facilitate the activation of inflammasome and subsequent cleavage of cytokine precursors. The second stimulus may come from the formation of mitochondrial reactive oxygen species (ROS), K+ efflux, membrane perturbations, or extracellular ATP, which induces oligomerization of NLRP3 and triggers assembly of the inflammasome (18, 21).

A number of recent reports demonstrate that the overactivation of NLRP3 inflammasome caused by various genetic abnormalities lead to development of colitis (22–24), whereas some other studies provide evidence that deficiency of NLRP3 and inflammasome related genes may induce more severe colitis in mice (25). Thus, the NLRP3 inflammasome may play a role in regulating a delicate immune balance in the colon, loss or overactivation of NLRP3 may both break down the balance and thus lead to the onset of colonic inflammation (14).

Regulation of NLRP3 inflammasome activation or its downstream cytokines may be a promising strategy for treating the IBD. A number of studies have shown that blockade of IL-1β or IL-1R signaling using antibodies can effectively ameliorate colon inflammation in Crohn’s disease and mouse model of colitis (7). However, antibody-based therapies are challenged by their high cost due to the high biologic dosage used in treatment and maintenance of diseases, which lead to a high economic burden to the patients (9). Moreover, the side effects of antibody-based therapies in treatment of IBD, such as serious infection and malignancy after administration of anti-TNF antibody, has long been observed and are still significant concerns of current IBD clinicians, which may limit the application of this type of therapies (26, 27). Thus, other alternative therapeutic strategies need to be developed for the intervention of IBD.

Previous studies have reported a number of small molecule inhibitors of NLRP3 inflammasome, such as MCC950 and CY-09 (28, 29), while their roles in IBD have not been evaluated. Studies in recent literature identified numerous new compounds derived from natural herbs may have a role in suppressing colitis in animal models through inhibiting inflammasomes. Here, we summarized the main findings of these studies and discussed the roles of these compounds in IBD models and the underlying mechanisms in inhibiting inflammasomes. This work may contribute to further mechanistical studies of these inhibitors in suppressing the activation of inflammasomes and may also be of benefit to future translational investigations.

## Traditional medicine derived compounds affect IBD *via* modulating the NLRP3 inflammasome

**Figure 2:**
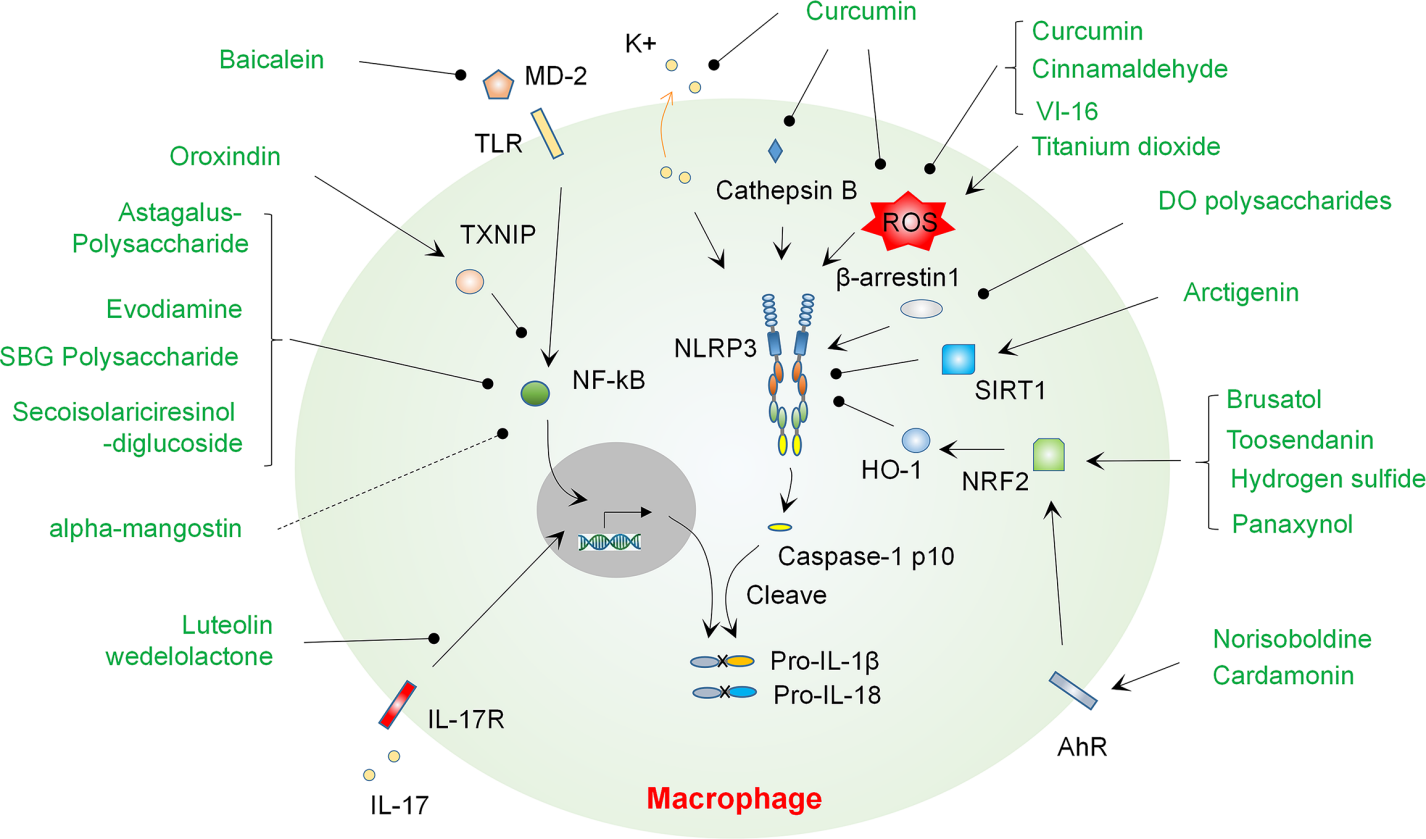
Natural compounds regulate NLRP3 inflammasome activation and progression of colitis *via* modulating various mechanisms. A number of natural compounds such as Baicalein suppress colitis by regulating priming of the NLRP3 inflammasome through modulating NF-κB activation, mainly *via* blocking MD-2 binding to TLR4 or inducing TXNIP expression. Blockade of IL-17R signaling by Luteolin or Wedelolactone may contribute to suppression of the priming signal of NLRP3 inflammasome. A handful of compounds including Curcumin can ameliorate colitis by suppressing the NLRP3 inflammasome *via* regulating ROS production, K+ efflux or cathepsin B release. Polysaccharides from DO may suppress β-arrestin-1 and thus inhibit the activation of NLRP3 inflammasome. A number of compounds such as Norisoboldine can promote activation of AhR and then activate NRF2 or SIRT1 signaling, which contribute the suppression of the NLRP3 inflammasome and amelioration of colitis. DO, *Dendrobium officinale.*.

Traditional medicine has long been used globally to treat a variety of human diseases, including infectious diseases, autoimmune diseases, cancers and IBD (30–34). Although the effects and exact mechanisms of traditional medicine are not yet fully validated and clearly addressed, it provides large numbers of compounds that may play a role in intervention of IBD. A variety of bioactive components derived from medicinal herbs have been found to play an inhibitory role in colitis *via* multiple mechanisms, and a few of them can prevent colitis at least partially by regulating the activation of inflammasomes *via* various mechanisms such as regulating NF-κB and ROS. Herein, we give an overview of these studies, discuss the cellular targets of compounds and their mechanisms, and provide an outlook for future studies (Summarized in **Figure 2** and **Table 1**).

**Table 1:** Summary of Natural Compounds that Regulates Inflammasomes in Colitis Models.

Bioactive Compound	Source	Target	Mechanism	Disease model	Impact to host	Toxicity Data	References
Astagalus Polysaccharide	*Astragalus membranaceus*	NF-κB	Inhibit NF-κB activation and expression of inflammasome components	DSS- colitis	Reduce DAI and histological injury scores	Not available	Tian et al. (35)
Oroxindin	*Astragalus membranaceus*	TXNIP, NF-κB	Promote expression of TXNIP and suppress NF-κB activation	DSS- colitis	Suppress macrophages infiltration and attenuate pathological changes in colonic tissue	Not available	Liu et al. (11)
Evodiamine	*Evodia rutaecarpa*	NF-κB	Inhibit NF-κB activation	DSS- colitis	Ameliorate mice body weight loss, DAI, colon length shortening, colonic pathological damage	Not available	Shen et al. (36)
SBG Polysaccharide	Scutellaria Baicalensis Georgi	NF-κB	Inhibit activation of NF-kB	DSS- colitis	Decrease DAI, colonic pathological damage, and reduce MPO activity	No observed toxicity (500 mg/ml, 0.4 ml/10 g p.o., 7 days in mouse)	Cui et al. (37)
DO polysaccharides	*Dendrobium officinale* (DO)	β-arrestin1	Inhibit β-arrestin1 expression	DSS-colitis	Decrease mortality, alleviate colonic pathological damage	No observed toxicity (200 mg/kg/day p.o. for 7 days in mouse)	Liang et al. (10)
Arctigenin	Fructus Arctii (Great Burdock Achene)	SIRT1	Suppress SIRT1 activation	DSS- colitis	Suppressed colon inflammation	Not available	Pu et al. (38)
Baicalein	Scutellaria baicalensis Georgi	MD-2	Suppress MD-2 binding to TLR4 and block TLR4/MyD88 Signaling	TNBS colitis	Decrease the activity of MPO and the expression of pro-inflammatory mediators.	Not toxic to RAW264.7 cells (200μM within 48h)	Luo et al. (12)
Cinnamaldehyde	Cinnamon	ROS	Prevent the production of ROS	DSS- colitis	Reduce loss of body weight, DAI, colon shortening and infiltration of inflammatory cells	Not toxic to RAW264.7 cells (100μM within 24h)	Qu et al. (39)
Flavonoid VI-16	Fruit and vegetable	mitochondria ROS	Reduce the mitochondrial ROS	DSS- colitis	Reduce colitis severity	Not available	Zhao et al. (40)
Curcumin	Curcuma longa species	K+ efflux, ROS and cathepsin B	Suppress K+ efflux, intracellular ROS formation and cathepsin B release	DSS- colitis	Suppress the colitis severity	Not available	Gong et al. (41)
Titanium dioxide	Food additive	ROS	Induce ROS production	DSS- colitis	Increase the severity of colitis	Not available	Ruiz et al. (42)
Luteolin and wedelolactone	Wedelia chinensis	IL-17 signaling pathway	Suppress genes in IL-17 signaling pathway	DSS- colitis	Suppress the colitis severity	Not available	Lin et al. (43)
Brusatol	Brucea javanica	NRF2	Activate NRF2	TNBS colitis	Attenuate diarrhea, colonic shortening, macroscopic damage and histological injury	Not toxic to RAW264.7 cells (200nM within 24h)	Zhou et al. (44)
Toosendanin	Melia toosendan Sieb et Zucc	NRF2/HO-1	Upregulate NRF2/HO-1 expression	DSS- colitis	Ameliaorate DAI, shortened colon length, pathological damage of the colon tissues	Not available	Fan et al. (45)
Hydrogen sulfide	Diet	NRF2	Upregulate NRF2 and Reduce ROS generation	DSS- colitis	Attenuate colitis severity, reduce colon shortening and colonic pathological damages	Not available	Qin et al. (46)
Norisoboldine	Radix Linderae	AhR	Activate AhR, elevated NRF2 and reduce level of ROS	TNBS colitis	Alleviate colitis related symptoms	Not toxic to THP-1 cells (30μM within 24h)	Lv et al. (13)
Cardamonin	Cardamom	AhR	Activate AhR/NRF2/NQO1 pathway	DSS-colitis, TNBS-colitis	Reduce colitis severity	Not toxic to THP-1 and BMDM cells (100μM within 24h)	Wang et al. (47)
Palmatine	A number of herbs	PINK1/ Parkin	Enhance the expression of PINK1 and parkin	DSS- colitis	Attenuate body weight loss and colon shortening, reduced DAI and histopathologic score	No observed toxicity (521 mg/kg/day, p.o.,90 days in mouse)	Mai et al. (48)
alpha-mangostin	A number of herbs	Not clear	Reduce NLRP3, caspase 1, IL-18, and IL-1β expression	LPS- colitis	Reduce severity of intestinal villi detachment, reduce congestion and hemorrhage, reduce epithelial cell nuclei deformation and the mitochondria swelling.	Not available	Yin et al. (49)
Secoisolariciresinol diglucoside	Lignans	NF-kB	Disruption of NF-kB activation and suppression of NLRP1 inflammasome	DSS- colitis	Attenuate the severity of colon inflammation and macrophage infiltration to the colon	Not toxic to RAW264.7 cells (25μM within 24h)	Wang et al. (50)
Apigenin	Plant and fruit	NLRP6	Reshape gut microbiota	DSS- colitis	Protect mice from colon damage	Not available	Radulovic et al. (51)
Panaxynol	Ginseng	NRF2	Activate NRF2 and reduce ROS	DSS- colitis	Reduce colitis severity	No observed toxicity (1mg/kg/day, p.o., 7 days in mouse)	Chaparala et al. (52)

DAI, Disease activity index; AhR, Aryl hydrocarbon receptor

### NF-κB

NF-κB is a transcription factor that plays a prominent role in diverse physiological processes including inflammatory responses. The activation of NF-κB promotes the production of pro-inflammatory cytokines in innate cells and regulates the activation and differentiation of lymphocytes. In the process of inflammasome activation, NF-κB provides the priming signal and promotes the transcription of inflammasome components, such as NLRP3 and Pro-IL-1β.

Recent studies found that a number of herbal compounds can affect the priming of inflammasome *via* regulating the activation of NF-κB. A good example for this type of compounds is oroxindin, a bioactive extract with anti-inflammatory activity derived from *Astragalus membranaceus* (AM), which is a medicinal plant wildly used in Chinese medicine for treatment of many human diseases (53, 54). Liu et al (11) found that oroxindin can suppress the activation of NF-kB signaling *via* inducing the expression of thioredoxin-interacting protein (TXNIP), an inhibitory regulator of NF-kB pathway (55). Considering the previously reported role of TXNIP in interacting and activating NLRP3 (56), this regulatory molecule may regulate inflammasome activation in both priming and activating steps. This may also explain the observation in Liu et al. study showing that the formation of NLRP3 inflammasome was also suppressed by oroxindin. The authors used a colitis model induced by dextran sulfate sodium (DSS), a sulfated polysaccharide that is toxic to colon epithelium and causes epithelial cell injury, the entry of luminal organisms or their products induce inflammatory responses in the lamina propria (57, 58). Their data showed that oroxindin can suppress macrophage infiltration to the colon and attenuated colon pathological changes. On this basis, the role of oroxindin in suppressing NLRP3 inflammasome function may mainly dependent on its inhibition on TXNIP associated NF-κB signaling. It should be noted that another compound extracted from AM, Astagalus Polysaccharide (AP), also showed anti-inflammatory effects (59, 60) and anti-colitis role *via* regulating the NLRP3 inflammasome. Its impact on inflammasome may be mediated by inhibiting the NF-κB pathway, since treatment with AP suppressed the expression of NLRP3, ASC, caspase-1, IL-18, and IL-1β (35). Interestingly, polysaccharide from *Scutellaria Baicalensis Georgi* (SBG) (37) also has anti-NF-κB effects, through which it inhibits NLRP3 inflammasome activation and mitigates DSS induced colitis.

Another compound that may play anti-colitis role through regulating NF-κB and the NLRP3 inflammasome is Evodiamine (EVO), a bioactive component with anti-inflammatory role (61) derived from *Evodia Rutaecarpa* (ER), which is a herbal plant that has been used clinically in treating some human inflammatory diseases, especially for headache and abdominal pain (62). To study the role of EVO in colitis, Shen et al (36) used murine DSS colitis model and found that treatment of EVO ameliorated DSS induced colonic symptoms, inhibited the production of pro-inflammatory cytokines such as TNF-α, IL-1β and IL-6, and restrain the activation of NLRP3 inflammasome. Regarding the mechanisms, they found that EVO can suppress activation of NF-κB and thus suppress the expression of inflammasome components.

### β-arrestin1

β-arrestin1 is a ubiquitously expressed protein that is identified initially as an inhibitor and adaptor of the G protein-coupled receptors with the purpose of regulating many immunological pathways and cell death (63, 64). It can also bind with a number of signaling molecules in MAPK pathways and thus regulate their activities in macrophages (64). Regarding its role in inflammasome activation, β-arrestin1 is required for full activation of NLRP3 and NLRC4 inflammasomes (65). Regulating β-arrestin1 may affect the development of colitis, in Liang et al.’s study (10), the authors studied the impact of polysaccharides extracts from *Dendrobium officinale* (DO) on colonic inflammation induced by DSS. DO is a widely used herb in traditional Chinese medical practice to treat gastrointestinal disorders. The data showed that polysaccharides from DO (DOPS) can inhibit β-arrestin1 expression and suppress activation of the NLRP3 inflammasome in both *in vivo* and *in vitro* experiments.

### Sirtuin 1 (SIRT1)

SIRT1 is a transcription factor that regulates various biological pathways. It can function as a histone deacetylase in the presence of nicotinamide adenine dinucleotide (NAD) (66). SIRT1 can suppress the production of pro-inflammatory cytokines by regulating deacetylation of NF-κB p65, thus it also affects the priming of NLRP3 inflammasome components. Moreover, SIRT1 may inhibit NLRP3 inflammasome activation by decreasing expression of CD40/CD40L (67) or suppressing oxidative stress (68). A recent study found that (38) SIRT1 may be inhibited by Arctigenin, one of the major bioactive components of *Fructus Arctii* (Great Burdock Achene), which is a herbal plant widely used in traditional Chinese medicine. It has been reported that Arctigenin has extensive pharmacological effects in a variety of diseases such as diabetes (69). Regarding its impact on colitis, Pu et al (38) found that Arctigenin can mitigate DSS induced colon inflammation *via* suppressing NLRP3 inflammasome. This effect was mediated by SIRT1, since knock-downing of SIRT1 decreased anti-inflammasome activity of Arctigenin.

### Myeloid differentiation protein-2 (MD-2)

MD-2 is a critical protein that assists the TLR4 receptor in sensing LPS (70). Thus, it may regulate the priming step of NLRP3 inflammasome activation. A study by Luo et al (12) found that Baicalein (5,6,7-trihydroxyflavone), a bioactive ingredient isolated from the root of SBG with potent anti-inflammatory and anti-cancer effects *via* multiple mechanisms (71, 72), may regulate the progression of colitis by targeting MD-2. The researchers employed a mouse model of colitis induced by intrarectally administration of 2,4,6-trinitrobenzene sulfonic acid (TNBS), a haptenic agent that renders colonic proteins immunogenic to the host immune system and drives an inflammatory response in the lamina propria. Baicalein can alleviate colitis severity induced by TNBS by inhibiting the activation of NLRP3 inflammasome and production of IL-1β in the colon. Further studies showed that the effect of baicalein may be associated with its role in blocking MD-2 binding with TLR4 and thus suppressing down-stream pathways, such as NF-kB and p38 MAPK. Thus, the impact of baicalein on the activation of the NLRP3 may be through regulating the priming signal, whereas if baicalein affects the activating signal of NLRP3 inflammasome need to be studied in future.

### K^+^ efflux, ROS production and cathepsin B release

K^+^ efflux, ROS production and cathepsin B release are the three major upstream signals that stimulate the activation step of the NLRP3 inflammasome formation. ROS is a term to describe a group of unstable molecules that contains oxygen and easily react with other molecules. The accumulation of ROS in cells may result in damage of DNA, RNA and protein, and ultimately cause cell death. The aberrant production of ROS is associated with pathogenesis of diverse inflammatory diseases and cancers.

Regulating these signals may affect inflammasome activation and colitis. For instance, Gong et al. (41) showed that curcumin, the principal curcuminoid of Curcuma longa species with anti-oxidative activity (73), can suppress K^+^ efflux, intracellular ROS formation and cathepsin B release, and thus inhibit NLRP3 inflammasome activation. Regarding the role of curcumin in development of colitis, it can attenuate DSS induced colitis symptoms. In addition, some clinical trials have demonstrated that curcumin is efficient and safe chemical for management of IBD (74).

Regulating the level of ROS may contribute to the progression of colitis. Qu et al (39) found that Cinnamaldehyde (CA) can inhibit colitis development by suppressing the NLRP3 inflammasome *via* regulating ROS. CA is a major bioactive ingredient with impressive antibacterial and anti-inflammatory activity derived from cinnamon (75, 76). The researchers showed that CA was effective in preventing DSS induced colitis associated conditions, it prevented activation of the NLRP3 inflammasome and production of pro-inflammatory cytokines in the colon. For the mechanisms, the authors found that CA can decrease the production of ROS and additionally suppress the phosphorylation of AKT, mTOR and COX_2_ in macrophages. Previous reports have demonstrated that ROS is an important regulator of the NLRP3 inflammasome (77–79), thus the anti-colitis and anti-inflammasome effects of CA may exert *via* down-regulating ROS. Another example on this direction is provided by the study of Zhao et al. (40). They studied the role of VI-16, a synthetic flavonoid compound, in colitis. Flavonoids are a group of bioactive polyphenols abundant in fruits and vegetables (80, 81). Previous studies have reported that a number of flavonoids affect the activation of inflammasome (82), but the mechanism is not fully clarified. Using DSS induced colitis model, Zhao et al. (40) showed that VI-16 administration protected mice from colon inflammation, and this protection was dependent on NLRP3 expression in hematopoietic cells. Regarding the mechanisms, VI-16 was able to reduce the level of mitochondrial ROS and thus block TXNIP-NLRP3 interaction.

An opposite example regarding the role of ROS in colitis is provided by the study of Ruiz et al (42), they studied the possible role of Titanium dioxide (TiO_2_) in colonic inflammation. TiO_2_ is a natural oxide of titanium that widely used in food products as additives or in pharmaceutical formulations, although a minor human health risk of TiO_2_ nanoparticles has been proposed (83). A number of recent studies have shown that TiO_2_ play a role in regulating autophagy, oxidative stress and apoptosis (84, 85). In Ruiz et al.’s study, the researchers found that oral administration of TiO_2_ nanoparticles increased the severity of colitis induced by DSS, TiO_2_ particles were taken up by intestinal epithelial cells and macrophages, and induced production of ROS, which then triggered the formation of the NLRP3 inflammasome. Hence, intake of food with TiO_2_ particles may worsen the disease for patients with colitis.

### IL-17 pathway

A number of recent studies showed that IL-17 pathway may act as a priming signal to promote expression of NLRP3 and IL-1β (86, 87). Thus, targeting IL-17 signaling may also affect the activation of NLRP3 inflammasome and contribute to progression of colitis. For instance, a study conducted by Lin et al (43) identified two compounds, wedelolactone and luteolin, from the bioactive fraction of *Wedelia chinensis* (WC) extracts, which has been proven to be effective in murine colitis model (88), and found that both compounds can suppress DSS induced colitis, and suppress the expression of NLRP3 and NLRP1 in the colon. Additionally, the inhibitory effects of luteolin on NLRP3 expression is mediated by suppressing genes in IL-17 signaling pathway, while the exact targets of luteolin and related mechanisms need to be identified in future.

### Nuclear factor erythroid 2-related factor 2 (Nrf2)/heme oxygenase-1 (HO-1)

Nrf2 is an important transcription factor that regulates an array of genes in modulating antioxidant and detoxifying systems. A number of previous studies showed that Nrf2 can suppress NLPR3 inflammasome activation by promoting expression of antioxidant genes, such as HO-1 (89), or inhibiting the expression of TXNIP (90). Studies by Zhou et al. (44, 91) found that colonic pathological changes induced by DSS or TNBS can be inhibited by Brusatol, a bioactive ingredient with anti-inflammatory effect (92) extracted from *Brucea javanica* (BJ), which is a medicinal plant used for treatment of ulcerative colitis (UC) in Chinese medicine (93). For the mechanisms, the authors showed that the compound can activate Nrf2 and suppress the NLRP3 inflammasome activation illustrated by decreased expression of IL-1β and IL-18 in the colon. Thus, Brusatol may exert its anti-inflammasome role by up-regulating Nrf2 mediated antioxidant activity.

Another compound that may suppress colitis and NLRP3 inflammasome through Nrf2/HO-1 pathway is Toosendanin (TSN), a triterpenoid and natural insecticide with anti-inflammatory activity derived from *Melia toosendan Sieb et Zucc* (MTSZ), which is traditional herbal medicine used as parasiticide of digestive tract and agricultural insecticide in China (94). For its role in colitis, Fan et al. (45) reported that TSN can attenuate DSS induced colonic inflammation *via* regulating the activation of NLRP3 inflammasome, the compound can also inhibit M1 macrophage polarization and production of pro-inflammatory cytokines and mediators in oxidative stress. These effects of TSN may be associated with its role in upregulating Nrf2/HO-1 expression in the colon and thus preventing M1 macrophage polarization and NLRP3 inflammasome activation.

Recent studies have provided evidence that high fat or high glucose diet may change the level of intracellular compounds such as Hydrogen sulfide (H_2_S) (95, 96)and alter the colonic homeostasis by regulating ROS production. H_2_S is a gaseous intracellular signal transducer that plays an anti-inflammatory effect in various diseases (97, 98). The role of H_2_S in IBD was evaluated by Qin et al (46) showing that H_2_S attenuated DSS induced colitis, H_2_S treated mice manifested reduced colon shortening and colonic pathological damages. H_2_S treatment inhibited expression of NLRP3 and cleavage of caspase-1 in the colon of mice. In *in vitro* studies, H_2_S can significantly inhibit NLRP3 inflammasome activation in bone marrow macrophages. For the mechanisms, the authors showed that H_2_S can disrupt Nrf2 activation, which may then affect intracellular ROS generation.

### Aryl hydrocarbon receptor (AhR)

AhR is a universally expressed transcription factor that can bind to a variety of structurally diverse ligands and regulates a range of biological processes such as metabolism of xenobiotic chemicals, immunity and stem cell differentiation (99). It has been shown that AhR can suppress NLRP3 inflammasome activation by inhibiting NLRP3 transcription (100). A study conducted by Lv et al (13) found that a herbal compound, Norisoboldine (NOR) can alleviate colon inflammation induced by TNBS by targeting AhR. NOR is a bioactive alkaloid purified from Radix Linderae (RL), which is the root of *Lindera strychnifolia*, traditional Chinese medicine used in treating various diseases. The researchers showed that NOR alleviated colitis and reduced the levels of NLRP3, cleaved caspase-1 and IL-1β in colons of the mice. In *in vitro* studies, NOR was shown to activate AhR, which then enhanced Nrf2 level and reduced production of ROS. Thus, the anti-colitis role of NOR is mediated by inhibiting the NLRP3 inflammasome activation *via* modulating AhR/Nrf2/ROS pathway.

Cardamonin is a chalcone derived from cardamon with anti-cancer activity, it also plays a role in anti-infection immunity and the development of inflammatory diseases. A recent study has shown that cardamonin can inhibit the NLRP3 inflammasome activation and thus attenuate experimental colitis induced by both DSS and TNBS (47). In this study, the researchers found that cardamonin can activate the AhR/Nrf2/NQO1 pathway, blocking this pathway can abolish the effect of cardamonin on NLRP3 inflammasome.

### Mitophagy

Mitophagy is an autophagic pathway that keeps the cell healthy by promoting the mitochondria turnover and preventing the accumulation of damaged mitochondria (101). It regulates the production of ROS and the release of mitochondria DNA. In mammals, mitophagy can be regulated by many factors such as PINK1 and parkin, whose deletion or mutation may cause impaired mitophagy and thereby leading to a variety of inflammatory diseases and cancers (102, 103). Previous studies have demonstrated that mitophagy is a negative regulator of NLRP3 inflammasome activation (104) and an important regulator of gut homeostasis (105). In a study conducted by Mai et al (48) showed that targeting mitophagy by palmatine, an isoquinoline alkaloid with potent anti-inflammatory and anti-bacteria effects purified from herbal plants (106), can protect the host from colitis. The authors showed that palmatine attenuated DSS induced colon pathology and reduced the level of MPO, IL-1β, TNF-α, and macrophage infiltration to the colon. In mechanistical studies, they found that palmatine suppressed NLRP3 inflammasome activation, an impact that can be blocked by mitophagy inhibitor Cyclosporin A or PINK1 siRNA. On this basis, the suppression of palmatine on colon inflammation was mediated by inhibiting the NLRP3 inflammasome *via* modulating PINK1/Parkin regulated mitophagy.

### Unknown targets

As described above, studies have identified the targets of many natural compounds in regulating the progression of colitis and the activation of the NLRP3 inflammasome, whereas, for some other compounds, the investigation that identifying their intracellular targets is still in progress. One of these compounds is Alpha-mangostin (alpha-MG), a natural xanthonoid derived from the fruit hull of mangosteen (*Garcinia mangostana* L.) that has anti-cancer activity (107, 108). Being a potent antioxidant, a previous study proved that alpha-MG can reduce LPS induced inflammatory responses in IEC-6 cells (109). Using a high throughput sequencing method, Yin et al (49) found that the genes regulated by alpha-MG were mainly related to inflammation and oxidative stress. Using an LPS induced IBD model in rat, they found that pretreatment of alpha-MG significantly reduced all the pathological changes caused by LPS including detachment of intestinal villi, deformation of intestinal epithelial cell nuclei and swelling of mitochondria, and these effects of alpha-MG was exerted by suppressing the expression of NLRP3, caspase 1, IL-18, and IL-1β.

## Herbal medicine that affects IBD *via* other inflammasomes

As described above, regulation of the NLRP3 inflammasome using herbal compounds can affect the development of colitis *via* multiple mechanisms, whereas previous studies have shown that other inflammasome forming NLRs such as NLRP1, NLRP6, NLRC4 and AIM2 are also involved in the pathogenesis of colonic inflammation (110–113). Thus, we searched the literature and found that several compounds affect the development of colitis by targeting NLRP1 and NLRP6. Secoisolariciresinol diglucoside (SDG), a bioactive compound derived from lignans, plays anti-inflammatory and antioxidant roles in various diseases (114, 115). A study performed by Wang et al (50) showed that SDG can attenuated colon inflammation and macrophage infiltration to the colon in a mouse model induced by DSS. Meanwhile, SDG can inhibit the activation of NLRP1 inflammasome and the production of pro-inflammatory cytokines including IL-1β, IL-18 and TNF-α in both colon tissue of DSS treated mice and RAW264.7 macrophages. The effect of SDG on NLRP1 inflammasome was partly dependent on disruption of NF-kB activation.

A previous study showed that apigenin, a flavone that has antioxidant effect and is involved in development of various diseases (116), can suppress DSS induced colitis *via* regulating the activation of NLRP6 inflammasome. In this study, Radulovic et al (51) found that found that mice were protected from DSS induced colitis after cohousing with apigenin treated animals. In contrast, deficiency of NLRP6 disrupted the protective effect of apigenin on the mice. Data of 16S rRNA sequencing showed that apigenin induced a composition change in gut microbiota, which was absent in NLRP6 deficient mice. In addition, the impact of apigenin on colitis was not affected by the absence of caspase-1/11 or ASC. Thus, these studies indicated an inflammasome independent mechanism of NLRP6 whereby apigenin reshaped gut microbiota through NLRP6 and thus protected mice against colitis.

## Herbal compounds that possibly affect colitis *via* regulating inflammasomes

Ginseng has been wildly used in treatment of a variety of human diseases including IBD. Studies have shown that the beneficial activities of ginseng are associated with its metabolites such as compound K (CK), 2-(S)-protopanaxatriol, Rh1, F1, and 20(S)-protopanaxadiol. Seong et al (117) showed that fermented wild ginseng (FWG) alleviated the severity of colitis induced by DSS and reduced the infiltration of macrophages in colonic tissue. FWG also inhibited the expression of IL-1β in LPS treated macrophages. In another study, researchers found that American ginseng extracts and the bioactive component, panaxynol, ameliorated colitis by activating Nrf2 pathway and thus reducing ROS production (52, 118). These evidences suggest that panaxynol may affect colitis by regulating the activation of an inflammasome. Ginsenoside Rf is another bioactive compound extracted from ginseng. A study by Ahn et al (119) reported that ginsenoside Rf can decrease the production of IL-1β, IL-6, TNF-a, NO, and ROS, mediators that are highly activated in IBD, in TNF-α stimulated intestinal epithelial cells and macrophages. Additionally, ginsenoside Rf suppressed TNF-α/LPS-induced NF-kB activity. Hence, ginsenoside Rf has potent intestinal anti-inflammatory effects and may have potential to treat IBD. The effect of ginseng extracts on IL-1β expression and ROS indicated a possible involvement of inflammasomes in the anti-colitis function.

*Indigo naturalis* (IN) is herbal medicine extracted from leaves and stems of plants and is a component of crude drugs used in treating many diseases including IBD (120). However, studies have shown that inappropriate application of IN can also aggravate colitis and pulmonary arterial hypertension, thus, using it topically has been suggested by researchers in application of IN in treatment of IBD (120). Another way that may reduce the side effects of IN is to identify the effective components in IN, since the effects of IN may be associated with the bioactive ingredients it contains. For instance, IN derived indigo, indirubin, 6-formylindolo [3,2-b] carbazole (FICZ), and indole-3-carboxaldehyde (IAId) can act as ligands and activate aryl hydrocarbon receptor (AhR), a pathway that can promote mucosal healing by regulating the IL-22 production from type-3 innate lymphocytes cells (120). Considering the inhibitory effect of AhR signaling on NLRP3 inflammasome activation (100), AhR ligands derived from IN may also have an impact on the NLRP3 inflammasome. Thus, the compounds affecting inflammasome regulators may modulate the progression of colitis *via* altering activation of inflammasomes. It is worthwhile to examine the roles of these compounds in a colitis model, which may contribute to the design of new anti-colitis strategies.

## Limitations and possible solutions

Traditional or alternative medicine has been used globally for centuries, the efficacy and safety of a variety of decoctions have been evaluated in large amounts of clinical cases. While the application of traditional medicine, especially the decoctions, is largely dependent on physician’s judgement to patients’ particular conditions, which to a great extent rely on physician’s knowledge and experience, and thus limits the wide application of traditional medicine. A decoction usually contains a number of different herbals or mineral materials for disease treatment, thus it may contain thousands of compounds with various effects. However, the compound composition of a particular herbal plant may largely rely on its growing environment such as soil conditions. Hence it is hard to exactly predict the integrated function of the compounds in a herbal decoction, some adverse events may occur in hospitals using traditional medicine (121). To overcome this problem, integrated analysis of the patients’ physiological condition and the functional compounds in an effective herbal decoction is necessary in future studies.

As mentioned previously, most of the herbal derived compounds, such as curcumin and some ginseng products, are water insoluble, unstable and inefficient in transiting across the physiological barriers, hence it is challenging to reach a concentration in the plasma or the affected tissues that is effective to treat a disease. A reported way that can improve the bioavailability of herbal compounds is combined administration with particular adjuvant compounds. For instance, studies decades ago had found that concomitant administration of piperine can largely improve the plasma concentration of curcumin (122). Similarly, a recent study showed that curcumin solubilized in essential turmeric oils showed substantially enhanced anti-inflammatory effects compared with curcumin alone in DSS-induced colitis (123). Further studies showed that some components contain in turmeric oils such as turmerones have anti-inflammatory role (124) and may the therapeutic benefits of curcumin in colitis model.

Another way that can effectively improve the bioavailability of herbal compounds is by using delivery-aid carriers. Accumulating evidences have shown that nanocarriers, such as lipid nanoparticles and liposomes, can efficiently increase the blood concentration of insoluble herbal compounds. For instance, Yang et al. (125) showed that polymer-based nanoparticles can significantly enhance curcumin delivery to the brain of mice. In another study, researchers found that nanoparticle encapsulation can increase the curcumin oral bioavailability for at least 9-fold compared with concomitant administration of curcumin with piperine (126). Therefore, application of nanoparticle-based carriers may be a promising approach for improving herbal compound bioavailability. Liposomes are vesicles formed by phospholipid bilayers (127), which can solubilize water insoluble compounds and transport these compounds into cells by membrane fusion. Many liposome-based drug delivery systems have been developed and showed enhanced bioavailability for delivery of a great number of water-insoluble herbal compounds in recent studies (128). For instance, a study by Telange et al. (129) showed that the formation of apigenin-phospholipid complex remarkably enhanced water solubility of apigenin for over 36-fold compared to pure apigenin.

Traditional medicine can be considered as a summary of experience from hundreds of ancient physicians in their medical practice, and it has been proven to be effective in many cases. Thus, it at least gives us a hint to discover compounds that are possibly effective to a particular disease. As discussed above, many compounds that prevent inflammation in colitis models described in this review are discovered in this way. According to the findings mentioned here, natural compounds purified from medicinal herbs or dietary materials are promising agents in attenuating colitis severity in animal models. However, their potential side effects on other organs and tissues, their physiological impacts on human cells have not been fully evaluated. Furthermore, the functional ingredients of some decoctions are not identified and the specificity of compounds on inflammasomes or other inflammatory signaling pathways are not clear. Additionally, the exact mechanisms whereby medicinal compounds regulate inflammasome activity are not fully addressed. On this basis, further studies are needed to address these questions focusing on evaluating or reducing side effects, determining drug specificity and working mechanisms, altering compound dosage or combination may be helpful to optimize the efficacy and safety of the compounds. With a satisfied answer of these questions, a clinical trial in IBD patients is a must before recommending the use of these compounds in clinical practice.

As mentioned above, compounds that are effective in studies of animal models for diseases may meet other challenges when applied in translational studies, especially the doses on an mg/kg basis proven to be safe and effective in animal studies sometimes do not display satisfactory outcomes after scaling of the doses based on body weight and applying in human clinical trials. This condition is primarily due to the varied pharmacokinetics in species with different biochemical systems (130, 131). To overcome this problem, U.S. Food and Drug Administration has developed a guidance in estimating the human equivalent dose (HED) for a compound that can act as maximum recommended starting dose (MRSD) in its initial clinical trial based on data derived from animal studies (https://www.fda.gov/regulatory-information/search-fda-guidance-documents/estimating-maximum-safe-sta). According to this guidance, the estimation of HED requires studies to determine the No Observed Adverse Effect Level (NOAEL) of a compound in multiple tested animal species. This requires data on systemic toxicology and others, such as bioavailability, metabolite profile and plasma level of the compound. With the availability of all these data, the conversion of NOAEL to HED needs to employ a scaling system based on body weight (mg/kg) or body surface area (mg/m^2^), a process that needs to consider multiple factors, such as the method of compound administration and NOAELs in different animal species. However, the *in vivo* data in majority of studies mentioned in this review did not provide sufficient details to estimate the MRSD of the tested compounds. Only four out of twenty-two studies provided *in vivo* toxicity data that obtained from IBD model induced in one species of animal (**Table 1**). Some of other studies did *in vitro* toxicity tests in a number of cell lines, such as RAW264.7 and THP-1. Thus, the current available data are not enough for the estimation of MRSDs for the compounds mentioned here. More extensive animal studies with multiple species that determine the NOAELs, toxicology, tolerability and pharmacokinetic profiles are necessary for the estimation of its MRSD in future investigation for compounds with potential clinical applications.

## Conclusion

This review summarized recent studies reporting the role and mechanisms of traditional herbal medicine or diet derived compounds in modulating colitis *via* regulating inflammasome activity in IBD models. These studies support a point of view that herbal or diet derived compounds may inactivate inflammasomes in the colon and thus attenuate colonic inflammation in animal models, or IBD patients in some cases, by targeting various inflammasome regulators as described above. Whereas the role of inflammasomes in the progression of colon inflammation is complicated since a number of previous studies using animals deficient in inflammasome components such as NLRP3 and caspase-1 proved that inflammasome function is also required in the maintenance of gut homeostasis (7). Thus, the activation of inflammasomes needs an appropriate regulation to keep a balanced immune response in the colon. Modulating inflammasome activity using bioactive compounds purified from traditional medicine or diet might be a promising strategy for treatment of IBD. Establishment of a cellular screening model using new identification technologies might be helpful to discover new compounds specifically targeting inflammasomes for treatment of IBD. A deep understanding of the pharmacological characters of the compounds in preventing inflammasomes may expediting the development of new strategies with higher efficiency for treatment of IBD and many other inflammatory diseases.

## Author contributions

QX performed the literature search, drew the figures, and wrote the first draft of the manuscript. WS, JZ, YM, and JB helped in manuscript preparation. SH, XZ, and LM revised and edited the final version of the manuscript. All authors contributed to the article and approved the submitted version.

## Funding

This study was supported in part by National Natural Science Foundation of China (32070919, 32170915 and 82172931), Natural Science Foundation of Jiangsu province (BK20201442), Jiangsu Specially-Appointed Professor and Start-up funds for young scientists of Nantong University (03083051).

## Conflict of interest

The authors declare that the research was conducted in the absence of any commercial or financial relationships that could be construed as a potential conflict of interest.

## Publisher’s note

All claims expressed in this article are solely those of the authors and do not necessarily represent those of their affiliated organizations, or those of the publisher, the editors and the reviewers. Any product that may be evaluated in this article, or claim that may be made by its manufacturer, is not guaranteed or endorsed by the publisher.
